# Diagnosis of advanced cervical cancer, missed opportunities?

**DOI:** 10.1186/s12905-022-01668-3

**Published:** 2022-03-30

**Authors:** Jérémie Mattern, Irène Letendre, Jeanne Sibiude, Cécile Pénager, Asma Jnifen, Fatoumata Souare, Sophie Ayel, Thuy Nguyen, Laurent Mandelbrot

**Affiliations:** 1 Assistance Publique-Hôpitaux de Paris, Service de Gynécologie-Obstétrique, Hôpital Louis Mourier, 178 rue des Renouillers, 92700 Colombes, France; 2 Université de Paris, Paris, France; 3 Inserm IAME 1137, Paris, France

**Keywords:** Cervical cancer, Advanced stage, Screening, Late diagnosis

## Abstract

**Background:**

Cervical cancer is common worldwide. Despite the existence of primary and secondary prevention strategies, the survival rate is decreasing in France due to an increasing proportion of advanced-stage cancer. Our objective was to determine the factors associated with a diagnosis of cervical cancer at advanced stages in an urban population in France.

**Methods:**

A retrospective study was conducted on all consecutive records of patients diagnosed with cervical cancer between January 2006 and December 2018 in a single center in Paris. The data collected were demographic characteristics, medical and gynecological history, circumstances of diagnosis, diagnostic and therapeutic management. The patients were divided into two groups according to the FIGO 2018 stage at diagnosis: group A stages IA1 to IB2 and group B advanced stages IB3 to IVB.

**Results:**

Among 96 patients who were diagnosed with cervical cancer, 25 (26%) were in group A and 71 (74%) in group B. Women in group B had less frequently received regular gynecological care than in group A (36% vs 84.2%, *p* < 0.001) and fewer had Pap test screening in the previous 3 years (30.4% vs 95.0%, *p* < 0.001). Parity greater than 3 was more frequent in group B (69.6% vs 42.9%, *p* = 0.031). The diagnosis was made during a routine examination or cervical smear in only 9.23% and 16.18% respectively in group B, versus 60% of cases in 45.82% of cases in group A (*p* < 0.001 and *p* = 0.003). Vaginal bleeding was observed in 85.29% in group B versus 36% in group A (*p* < 0.001). Histological type was squamous cell carcinoma 87.32% of group B and 56% of group A (*p* < 0.001).

**Conclusion:**

Diagnosis of cervical cancers at advanced stages occurred mostly in women who did not benefit from the recommended screening. Universal access to screening is necessary for the prevention and early treatment of cervical cancer.

## Background

Cervical cancer is the fourth most common cancer [1] in women worldwide and the eleventh in France [1], with 3023 new cases and 1102 deaths yearly [2]. Cervical cancer is due to persistent infection with oncogenic subtypes of human papillomaviruses (HPV) [3]. This allows for primary prevention with anti-HPV vaccination. The progression from low-grade squamous intraepithelial lesions to invasive cancer occurs over a period of several years, thus allowing for secondary prevention. Cervical screening by cytology or HPV testing is used to detect lesions of the cervix and treat if high-grade lesions appear [3, 4].

Until recently, screening for cervical cancer in France was based on individual (or spontaneous) initiative. All women aged 25 to 65 were offered cervical cytology testings, yearly and then every 3 years after 2 negative tests [5].

While these primary and secondary prevention strategies have led to a decrease in the incidence, cervical cancer is one of the only cancers for which the 5-year survival rate is decreasing in France [6]. One of the main prognostic factors is the stage when the disease is discovered. Cervical cancers that are less than 4 cm (up to the FIGO IB2 stage, in the 2018 classification [7]), defined as localized, have a fairly good prognosis (more than 90% survival at 5 years) whereas tumors larger than 4 cm (FIGO IIa stages and beyond) have a 5-year survival rate of only 50% [7–10]. Localized cancers are eligible for immediate surgical management, whereas advanced stages require radio-chemotherapy.

The objective of this study was to analyze the factors associated with the diagnosis of cancer at an advanced stage.

## Methods

We performed a retrospective study on medical records in the department of Obstetrics and Gynecology of Louis-Mourier Hospital, a university hospital center of the Assistance Publique-Hôpitaux de Paris (APHP), near Paris, France. All consecutive cases with a diagnosis of cervical cancer were identified over the period 2006 to 2019 from pathology reports on cervical biopsies or surgical samples. The data were extracted from multidisciplinary tumor board files, computerized medical records and paper archives.

The study was approved by the French database security commission (Commission Nationale Informatique et Liberté) and by the Institutional Review Board (Comité d’Evaluation de l’éthique des projets de Recherche Biomédicale, IRB00006477: N°2019–038 January 31, 2020).

The patients were classified into two groups: those diagnosed with cervical cancer at a localized stage (FIGO stage IA1 to IB2) (group A) and those diagnosed with locally advanced or advanced cancer (FIGO stage IB3 to IV) (group B). We chose this classification because the first line of treatment changes beyond stage 1B2 [8, 9]. Patients whose files were partly or completely unavailable were excluded, as well as patients who refused access to their data for research purposes.

### Variables studied

Demographics and medical history included age, ethnicity, parity and gestation, Human immunodeficiency virus (HIV) status, smoking, psychiatric history, gynecological history (ectopic pregnancy, sexually transmitted infections), professional activity and social deprivation.

Access to care was assessed by whether the patient had regular gynecological examinations (as declared by the patient) and/or a cervical cytology test within the past 3 years. The circumstances of diagnosis were classified as: during a routine gynecological examination, by cytology screening, or due to symptoms such as spontaneous or post coital metrorrhagia. Clinical findings at the time of diagnosis were general symptoms (weight loss, pain), as well as the appearance of the cervix at the time of cancer diagnosis: normal appearance, budding, gross erosion, ulcer or necrosis. The histological type of cancer was classified as squamous cell carcinoma or adenocarcinoma.

The results of pretreatment evaluation PET and MRI and the presence of metastases at the time of diagnosis were noted for each patient as well as the therapies which were used, ie. surgery, radiotherapy, brachytherapy, chemotherapy. We checked for consistency between the stage at diagnosis and care according to the Assistance Publique-Hôpitaux de Paris (APHP) and European Society of Gynaecological Oncology (ESGO) guidelines at the time the woman was treated [8, 9].

### Statistical analysis

Qualitative variables were compared between groups using a Chi^2^ test or a Fisher test. The quantitative variables were analyzed by Student's t-test. The statistical significance threshold used was *p* < 0.05. The data was analyzed using Stata 14.0 software.

## Results

Between 2006 and 2019, 116 patients were diagnosed with cervical cancer in our department. After excluding 20 cases with missing files, we included 96 patients among whom 25 (26%) had localized cancer and were included in group A and 71 (74%) had advanced cancer and were included in group B.

### Patients’ characteristics (Table 1)

The socio-demographic characteristics did not differ significantly between the two groups, in particular age, professional activity or social deprivation. Parity above 3 was more frequent in group B than in group A (69.64% vs 42.86%, respectively, *p* = 0.03).

**Table 1:** Demographic characteristics, history and gynecological care according to diagnosis of cervical cancer at advanced (group B) versus localized (group A) stage

	Group A N = 25	Group B N = 71	*p*-value
Age, years (mean, SD)	52.4 (38–66.8)	51.7 (37.8–63.6)	
*Geographic origin*			0.55
France	3/13 (23.08%)	14/35 (40%)	
Maghreb	6/13 (46.15%)	13/35 (37.14%)	
Other	4/13 (30.77%)	8/35 (22.86%)	
Currently employed	4 /11 (36.36%)	15/33 (45.45%)	0.60
Psychiatric history	2/24 (8.33%)	9/63 (14.29%)	0.37
History of pelvic inflammatory disease or ectopic pregnancy	2/25 (8%)	3/63 (4.76%)	0.44
Current smoking	5/17 (29.41%)	10/33 (30.30%)	0.95
Social deprivation	3/15 (20%)	8/32 (25%)	0.51
Living with HIV	1/25 (4%)	4/65 (6.15%)	0.57
Parity > 3	9/21 (42.86%)	39/56 (69.64%)	0.03
Regular gynecological follow-up	16/19 (84.21%)	18/50(36%)	< 0.001
Cervical cytology in the past 3 years	19/20 (95.00%)	17/56 (30.36%)	< 0.001

Percentages are the frequency within each group, denominators for available data within the group

Regular gynecological follow-up was less frequent in group B (36% vs 84.21% in group A, *p* < 0.001) as well as the presence of cervical cytology in the past 3 years (30.36 vs 95%, respectively, *p* < 0.001).

The absence of cervical cytology in the past 3 years was not associated with smoking (*p* = 0.31), living with HIV (*p* = 0.33), working (*p* = 0.17) or social deprivation (*p* = 0.27).

### Circumstances of cancer diagnosis (Table 2)

In group B, the cervical cancer was discovered by routine examination or by cervical cytology in only 9.23% and 16.18% of cases, respectively, whereas in group A the proportions were 60% and 45.82%, respectively (*p* < 0.001 and *p* = 0.003). Of the total number of patients who had an abnormal cytology, 16/22 required colposcopy. Spontaneous vaginal bleeding occurred in 85.29% of cases in group B (vs 36% in group A, *p* < 0.001), but post-coital bleeding, although more suggestive of cervical cancer, was noted in just 11 patients, with no significant difference between the two groups. Cervical cancer was discovered with global deterioration in 31.34% of patients in group B.

**Table 2:** Circumstances of diagnosis of cervical cancer at advanced (group B) versus localized (group A) stage

	Group A N = 25	Group B N = 71	*p*-value
Routine examination	15/25 (60%)	6/65 (9.23%)	< 0.001
Cervical cytology screening	11/24 (45.82%)	11/68 (16.18%)	0.003
Colposcopy for the diagnosis	16/25 (64%)	24/57 (42.11%)	0.22
Vaginal bleeding	9/25 (36%)	58/68 (85.29%)	< 0.001
Post coital vaginal bleeding	2/25 (8%)	9/68 (13.24%)	0.39
*Appearance of cervix*			0.04
Normal appearance	5/24 (20.83%)	5/67 (7.46%)	
Budding	2/24 (8.33%)	24/67 (35.82%)	
Gross erosion, ulcer	13/24 (54.17%)	26/67 (38.81%)	
Local induration	3/24 (12.50%)	8/67 (11.94%)	
Necrosis	1/24 (4.17%)	4/67 (5.97%)	
General symptoms	0/25 (0%)	21/67 (31.34%)	0.001

Percentages are the frequency within each group, denominators for available data within the group

At initial examination, the cervix appeared normal in 7.14% in group B versus 20.83% of cases in group A; there was budding in more than a third of cases in group B.

### Disease staging (Table 3)

By definition, there were marked differences between the two groups in local extension and metastases.

**Table 3:** Findings of the work-up of cervical cancers at advanced (group B) versus localized (group A) stage

	Group A N = 25	Group B N = 71	*p*-value
Normal vaginal examination	9/21 (42.86%)	1/56 (1.79%)	< 0.001
*PET scan findings*			< 0.001
Local hypermetabolism	14/15 (93.33%)	14/52 (26.92%)	
Hypermetabolism extended in pelvis	1/15 (6.67%)	31/52 (59.62%)	
Hypermetabolism beyond pelvis	0/15 (0%)	7/52 (13.46%)	
*MRI findings*			< 0.001
Cervical lesion < 4 cm	13/21 (61.90%)	3/63 (4.76%)	
Cervical lesion > 4 cm	0/21 (0%)	7/63 (11.11%)	
Extension to parametrium and/or pelvic lymph nodes	3/21 (14.29%)	37/63 (58.73%)	
Extension to parametrium and pelvic, iliac and para-aortic lymph nodes	0/21 (0%)	8/63 (12.70%)	
Extension to bladder. Ureters or rectum	0/21 (0%)	8/63 (12.70%)	
No MRI lesions	5/21 (23.81%)	0/63 (0%)	
Distant metastases	0/22 (0%)	11/51 (21.57%)	0.013
*Pathology*			
Squamous cell carcinoma	14/25 (56%)	62/71 (87.32%)	0.001
Adenocarcinoma	11/25 (44%)	9/71 (12.68%)	0.001

Percentages are the frequency within each group, denominators for available data within the group

Pathology findings also differed, with squamous cell carcinoma in 56% of group A patients versus 87.32% of group B (*p* < 0.001).

### Treatments (Table 4)

The management of patients was decided by the multidisciplinary cancer team according to current guidelines, as updated during the study period. Over 90% of patients in group B received chemotherapy, mostly in association with radiation therapy. The general approach was to offer hysterectomy in advanced cases following radiation and chemotherapy when possible, but the decision to perform surgery became more restrictive over time. Thus only 34.8% of patients in group B had a hysterectomy versus 100% of patients in group A.

**Table 4:** Treatment for cervical cancer at advanced (group B) versus localized (group A) stage

	Group A N = 25	Group B N = 71	*p*-value
Total hysterectomy with adnexectomy	11/25 (44%)	9/66 (13.64%)	< 0.001
Total hysterectomy with adnexectomy + lymph node dissection	14/25 (56%)	14/66 (21.21%)	
No surgery	0/25 (0%)	43/66 (65.15%)	
Chemotherapy	6/24 (25%)	64/70 (91.43%)	< 0.001
Radiation therapy	8/24 (33.33%)	53/66 (80.3%)	< 0.001
Brachytherapy	11/24 (45.83%)	24/60 (40%)	0.62

Percentages are the frequency within each group, denominators for available data within the group

## Discussion

### Main findings

We found that lack of gynecological follow-up and cytological screening were associated with a diagnosis of cervical cancer at advanced stages. Most of the advanced-stage cancers were discovered following symptoms, in particular vaginal bleeding, as reported in other studies [11]. It should be noted that the cervix was clinically abnormal in over 75% of cases in group A, confirming the importance of screening and systematic clinical examinations to diagnose asymptomatic cancers at an early stage [3]. Also, we observed a higher proportion of adenocarcinomas in cases diagnosed at advanced stages.

We found no association between most socio-demographic variables such as age, ethnic origin, and social deprivation, and diagnosis at advanced stages. Contrary to our hypothesis that socially and economically disadvantaged women would have poorer access to care, this was not the case in our population regarding cytological screening. This contrasts with findings from the United States [12], which may be related to the differences in health care coverage between countries. In a survey by the French Public Health agency [13]), the factors associated with lack of cervical screening were an age above 50 years, disabled status, alcohol or opioid abuse, chronic diseases including obesity, diabetes, HIV infection, hepatitis and mental illness, as well as living in disadvantaged geographic areas. Our study did not have the power to investigate these factors. Parity was significantly associated with late diagnosis. The literature suggests that there are pathophysiological reasons for this association [14], because the hormonal changes during pregnancy alter the epithelial junction, the transformation zone which is most vulnerable to HPV infection. In addition, this junction is maintained in the exocervix longer in multiparous women. We found no differences between groups regarding smoking and HIV status, which are known to be risk factors for cervical cancer.

### Strengths and weaknesses

Most patients were cared for entirely in our center from the first visit, through diagnostic and staging procedures, therapy and comprehensive follow-up. This decreases the risk of recruitment bias in some dedicated pelvic cancer centers where patients are referred after diagnosis and assessment. Our recruitment was through gynecologic clinics as well as emergency room, thus closer to a population-based study. Also, the number of patients was relatively large for a single-center study.

The main weaknesses of our study are inherent to its retrospective nature, including potential selection bias and missing data. These were numerous for certain socio-demographic variables and social deprivation was not assessed with a systematic scale, such as the EPICES score [15, 16], because not all of the variables were routinely collected. The power was also limited, so we could not perform a multivariate analysis.

### Interpretation in view of the literature

In previous studies [17–21], mainly from Africa, where health infrastructures for screening and prevention differ, the factors associated with a diagnosis at an advanced stage of cervical cancer were high parity as in our study, and also low education level, long distance from the health center, and young age at first sexual intercourse, variables which were not available in our study, as well as living with HIV. The number of women with HIV in our population was insufficient to conclude, but it should be noted that we offer yearly gynecologic visits with cervical screening for these patients in our center.

In France, the coverage of cervical cytology was estimated in 2016 at 61.9% [22]. It was recommended every 3 years for women between 25 and 65 years of age. However, despite information campaigns on screening and the relative accessibility of gynecological follow-up by general practitioners, midwives or gynecologists, in the office or hospital, a large proportion of patients do not access such care.

In our center there is a structured network, with close collaboration allowing physicians and midwives to refer patients to our cervical diseases clinic through a dedicated channel, with access to colposcopy for low grade lesions within 1 month and for high grade lesions within 2 weeks of the cytology results. None of the patients diagnosed at an advanced stage were referred by the cervical diseases channel. This indicates that the high-grade lesions diagnosed within the center were treated appropriately with LEEP and ablative therapy and followed up properly, preventing progression to advanced stages. It is therefore essential to reinforce training for general practitioners, midwives, and healthcare providers in general.

Nevertheless, the performance of cytological screening is imperfect, with a sensitivity of only 58% [23]. Because the HPV subtype is highly associated with cervical transformations and cancer, and because the reproducibility of HPV testing is better than for cytology, the most recent guidelines recommend HPV testing as the first line of screening for all women between ages 30 and 64, with cytologic screening maintained in women from ages 24 to 29 [24]. Also, whereas the incidence of squamous carcinoma of the cervix has declined in countries with cytologic screening, the proportion of adenocarcinoma of the cervix has increased [25], which is less amenable to prevention through cervical screening by cytology [26].

## Conclusion

Non-engagement in care or screening stands out as the main factor for cervical cancer being diagnosed at advanced stages. This should encourage us to better identify missed opportunities for prevention and to take action on these factors. Guidelines are increasingly recommending screening with HPV testing beyond the age of 30^27^. Self HPV testing or alternative testing methods should be offered to persons who are not attending medical visits. Screening should be offered free of charge with an individual outreach to women by mail, as recently introduced in France. Simple messages remain to be communicated more effectively in mass information campaigns, and by systematic discussion about screening and vaccination during all types of medical visits.

## Data Availability

The datasets used and analyzed during the current study are available from the corresponding author on reasonable request.

## Abbreviations

HPV: Human papillomaviruses
HIV: Human immunodeficiency virus
EPICES: Evaluation de la précarité et des inégalités de santé dans les Centres d’examens de santé
APHP: Assistance Publique-Hôpitaux de Paris
ESGO: European Society of Gynaecological Oncology

## References

[1] Bray F, Ferlay J, Soerjomataram I, Siegel RL, Torre LA, Jemal A (2018). Global cancer statistics 2018: GLOBOCAN estimates of incidence and mortality worldwide for 36 cancers in 185 countries. CA Cancer J Clin.

[2] Binder-Foucard F, Bossard N, Delafosse P, Belot A, Woronoff AS, Remontet L (2014). Cancer incidence and mortality in France over the 1980–2012 period: solid tumors. Rev DÉpidémiologie Santé Publique.

[3] Cohen PA, Jhingran A, Oaknin A, Denny L (2019). Cervical cancer. The Lancet.

[4] Schiffman M, Castle PE (2005). The promise of global cervical-cancer prevention. N Engl J Med.

[5] En R, Publique S. État Des Lieux et Recommandations Pour Le Dépistage Du Cancer Du Col de l’utérus En France. 2010.

[6] Ribassin-Majed L, Le Teuff G, Hill C (2017). La fréquence des cancers en 2016 et leur évolution. Bull Cancer (Paris).

[7] Matsuo K, Machida H, Mandelbaum RS, Konishi I, Mikami M (2019). Validation of the 2018 FIGO cervical cancer staging system. Gynecol Oncol.

[8] Référentiel Cancers Du Col Utérin. 2016.

[9] Cervical Cancer Pocket Guidelines.

[10] Kosary CL. FIG0 Stage, histology, histologic grade, age and race as prognostic factors in determining survival for cancers of the female gynecological system: an analysis of 1973–87 SEER Cases of Cancers of the Endometrium, Cervix, Ovary, Vulva, and Vagina KEY WORDS: Cox Proportional Hazards Model, Survival, Survival Rate, Cancer, All Sites 32 Kosary, vol. 10. 10.1002/ssu.2980100107 8115784

[11] Stapley S, Hamilton W (2011). Gynaecological symptoms reported by young women: examining the potential for earlier diagnosis of cervical cancer. Fam Pract.

[12] Olusola P, Banerjee HN, Philley JV, Dasgupta S (2019). Human papilloma virus-associated cervical cancer and health disparities. Cells.

[13] SPF. Caractérisation des femmes ne réalisant pas de dépistage du cancer du col de l’utérus par frottis cervico-utérin en France. Numéro thématique. Vers la généralisation du dépistage organisé du cancer du col de l’utérus. Accessed 29 Nov 2021. https://www.santepubliquefrance.fr/maladies-et-traumatismes/cancers/cancer-du-col-de-l-uterus/caracterisation-des-femmes-ne-realisant-pas-de-depistage-du-cancer-du-col-de-l-uterus-par-frottis-cervico-uterin-en-france.-numero-thematique.-vers

[14] Muñoz N, Franceschi S, Bosetti C (2002). Role of parity and human papillomavirus in cervical cancer: the IARC multicentric case-control study. Lancet Lond Engl.

[15] Labbe E, Moulin JJ, Gueguen R, Sass C, Chatain C, Gerbaud L (2007). Un indicateur de mesure de la précarité et de la «santé sociale»: le score EPICES. Rev Ires.

[16] Sass C, Guéguen R, Moulin JJ (2006). Comparaison du score individuel de précarité des Centres d’examens de santé, EPICES, à la définition socio-administrative de la précarité. Sante Publique (Bucur).

[17] Berraho M, Obtel M, Bendahhou K (2012). Sociodemographic factors and delay in the diagnosis of cervical cancer in Morocco. Pan Afr Med J.

[18] Stewart TS, Moodley J, Walter FM (2018). Population risk factors for late-stage presentation of cervical cancer in sub-Saharan Africa. Cancer Epidemiol.

[19] Dunyo P, Effah K, Udofia EA (2018). Factors associated with late presentation of cervical cancer cases at a district hospital: a retrospective study. BMC Public Health.

[20] Nassali MN, Tadele M, Nkuba RM, Modimowame J, Enyeribe I, Katse E (2018). Predictors of locally advanced disease at presentation and clinical outcomes among cervical cancer patients admitted at a tertiary hospital in Botswana. Int J Gynecol Cancer.

[21] Mlange R, Matovelo D, Rambau P, Kidenya B (2016). Patient and disease characteristics associated with late tumour stage at presentation of cervical cancer in northwestern Tanzania. BMC Womens Health.

[22] InCa. Généralisation du dépistage du cancer du col de l’utérus/étude médico-économique/Phase 1—Ref : APDEPCCU16. Accessed 25 June 2019. https://www.e-cancer.fr/Expertises-et-publications/Catalogue-des-publications/Generalisation-du-depistage-du-cancer-du-col-de-l-uterus-etude-medico-economique-Phase-1

[23] Fahey MT, Irwig L, Macaskill P (1995). Meta-analysis of Pap test accuracy. Am J Epidemiol.

[24] PUBLIC HEALTH GUIDELINES SUMMARY Evaluation of Human Papillomavirus (HPV) tests for primary screening of precancerous and cancerous lesions of the cervix and the role of P16/Ki67 dual immunostaining; 2019.

[25] Islami F, Fedewa SA, Jemal A (2019). Trends in cervical cancer incidence rates by age, race/ethnicity, histological subtype, and stage at diagnosis in the United States. Prev Med.

[26] Renshaw AA, Mody DR, Lozano RL (2004). Detection of adenocarcinoma in situ of the cervix in Papanicolaou tests: comparison of diagnostic accuracy with other high-grade lesions. Arch Pathol Lab Med.

[27] HAS. Évaluation de la recherche des papillomavirus humains (HPV) en dépistage primaire des lésions précancéreuses et cancéreuses du col de l’utérus et de la place du double immuno-marquage p16/Ki67.

